# Supplementary material for: Stability of extemporaneously prepared sitagliptin phosphate solution
Source: PLoS One. 2022 Mar 16;17(3):e0262068. doi: 10.1371/journal.pone.0262068 (PMC8926268; doi:10.1371/journal.pone.0262068)
Supplement: Attachment — Submitted filename: Comment Sheet.docx [file pone.0262068.s001.docx]

Que.1: How you have selected a different formulation ingredients and ratio?

Que.2: Have you taken UV Spectrum for selection of wavelength in SiP solution? Is there same or different?

Que.3: Which guideline follow for the evaluation of stability studies of SiP solution?

Que.4: How you have evaluated organoleptic properties of SiP oral liquid formulation?

Que.5: Have you develop HPLC method or cite any other research article method? Describe it.
