# Supplementary material for: Stability of extemporaneously prepared sitagliptin phosphate solution
Source: PLoS One. 2022 Mar 16;17(3):e0262068. doi: 10.1371/journal.pone.0262068 (PMC8926268; doi:10.1371/journal.pone.0262068)
Supplement: Attachment — Submitted filename: Response Letter 15-11-2020.docx [file pone.0262068.s002.docx]

**Response Letter**

**Date: 15-11-2020**

**Girish Sailor
Academic Editor**

**Subject:** Revision of manuscript entitled " Stability of extemporaneously prepared Sitagliptin Phosphate Solution "

**Submission number:** PONE-D-20-27192

Below you can find The answer to the inquiries raised by the Editorial office (Journal requirement) and reviewers.

I. **Journal Requirements:**

 When submitting your revision, we need you to address these additional requirements.

 1. Please ensure that your manuscript meets PLOS ONE's style requirements, including those for file naming. The PLOS ONE style templates can be found at

<https://journals.plos.org/plosone/s/file?id=wjVg/PLOSOne_formatting_sample_main_body.pdf> and

<https://journals.plos.org/plosone/s/file?id=ba62/PLOSOne_formatting_sample_title_authors_affiliations.pdf>

- Done

2.Thank you for stating the following in the Acknowledgments Section of your manuscript:

[The authors would like to acknowledge An-Najah National University for its support]

We note that you have provided funding information that is not currently declared in your Funding Statement. However, funding information should not appear in the Acknowledgments section or other areas of your manuscript. We will only publish funding information present in the Funding Statement section of the online submission form.

I am sorry for this inconvenient, I would like to state that I have no funding for this project, it is only a research effort between my institution and Pharmacare laboratories only for academic purposes and there are no any commercial benefits for any of the authors or institutions.

Please remove any funding-related text from the manuscript and let us know how you would like to update your Funding Statement. Currently, your Funding Statement reads as follows:

 [The author(s) received no specific funding for this work]

 Please include your amended statements within your cover letter; we will change the online submission form on your behalf.

Thank you very much and I am sorry for this inconvenient. Also, I would like to state that I have no funding for this project, it is only a research effort between my institution and Pharmacare laboratories only for academic and public issues and there are no any commercial benefits for any of the authors or institutions.

 3. PLOS requires an ORCID iD for the corresponding author in Editorial Manager on papers submitted after December 6th, 2016. Please ensure that you have an ORCID iD and that it is validated in Editorial Manager. To do this, go to ‘Update my Information’ (in the upper left-hand corner of the main menu), and click on the Fetch/Validate link next to the ORCID field. This will take you to the ORCID site and allow you to create a new iD or authenticate a pre-existing iD in Editorial Manager. Please see the following video for instructions on linking an ORCID iD to your Editorial Manager account: <https://www.youtube.com/watch?v=_xcclfuvtxQ>

 Done

4.Thank you for stating the following in the Competing Interests section:

[The authors have declared that no competing interests exist].

We note that one or more of the authors are employed by a commercial company: Pharmacare PLC

1. Please provide an amended Funding Statement declaring this commercial affiliation, as well as a statement regarding the Role of Funders in your study. If the funding organization did not play a role in the study design, data collection and analysis, decision to publish, or preparation of the manuscript and only provided financial support in the form of authors' salaries and/or research materials, please review your statements relating to the author contributions, and ensure you have specifically and accurately indicated the role(s) that these authors had in your study. You can update author roles in the Author Contributions section of the online submission form.

- These authors at Pharmacare company played a pure academic rule in this manuscript such as carrying analytical experiments.

  Please also include the following statement within your amended Funding Statement.

“The funder provided support in the form of salaries for authors [insert relevant initials], but did not have any additional role in the study design, data collection and analysis, decision to publish, or preparation of the manuscript. The specific roles of these authors are articulated in the ‘author contributions’ section.”

- An-Najah National University provide support in the form of salaries to the following authors:
  - AN Z and
  - N J
- Pharmacare PLC provide support in the form of salaries to the following authors:
  - Y Z
  - A K
  - M G
- No salary from any for the following authors (they were students at the time of conducting the project:
  - T A S
  - S S
  - La O

If your commercial affiliation did play a role in your study, please state and explain this role within your updated Funding Statement.

- Not applicable

 2. Please also provide an updated Competing Interests Statement declaring this commercial affiliation along with any other relevant declarations relating to employment, consultancy, patents, products in development, or marketed products, etc.

- Not applicable

Within your Competing Interests Statement, please confirm that this commercial affiliation does not alter your adherence to all PLOS ONE policies on sharing data and materials by including the following statement: "This does not alter our adherence to  PLOS ONE policies on sharing data and materials.” (as detailed online in our guide for authors <http://journals.plos.org/plosone/s/competing-interests>) . If this adherence statement is not accurate and  there are restrictions on sharing of data and/or materials, please state these. Please note that we cannot proceed with consideration of your article until this information has been declared.

- Not applicable

Please include both an updated Funding Statement and Competing Interests Statement in your cover letter. We will change the online submission form on your behalf.

- Done

Please know it is PLOS ONE policy for corresponding authors to declare, on behalf of all authors, all potential competing interests for the purposes of transparency. PLOS defines a competing interest as anything that interferes with, or could reasonably be perceived as interfering with, the full and objective presentation, peer review, editorial decision-making, or publication of research or non-research articles submitted to one of the journals. Competing interests can be financial or non-financial, professional, or personal. Competing interests can arise in relationship to an organization or another person. Please follow this link to our website for more details on competing interests: <http://journals.plos.org/plosone/s/competing-interests>

I declare that no author has competing of interest in this manuscript.

 5.We noticed you have some minor occurrence of overlapping text with the following previous publication, which needs to be addressed:

- <https://insights.ovid.com/eujhph/201705000/01735171-201705000-00005>

In your revision ensure you cite all your sources (including your own works), and quote or rephrase any duplicated text outside the methods section. Further consideration is dependent on these concerns being addressed.

- Done

 Answer to reviewers’ comments

II. **Reviewers comments**

**Reviewer 1**

Reviewer #1: The above manuscript describes a method to evaluate the Stability of Extemporaneously Prepared Sitagliptin Phosphate Solution for patients with swallowing difficulties or need feeding tubes to take medicines. The research topic is interesting; however, it should be noted out that the manuscript exhibits many shortcomings and errors. Some are summarized below:

I would like to thank the reviewer for his comments that I appreciate and I believe they will raise the quality of the manuscript. The whole manuscript is now revised and all his/her comments are now answered appropriately.

Abstract: Line 31, Replace the word “champers” with “chambers”

Done
Abstract: Line 34, Replace the word “validates” with “validated”

Done
Introduction: Line 55, Delete the word “phosphate”

Done
Introduction: Line 59, The word “Mellitus” should be written as mellitus

Done
Materials and Methods: Line 82-85, Repetition of the word “Pharmacare PLC”

Done
Materials and Methods: Line 93, Incubator was used as stability chamber for stability studies. How temperature and humidity can be varied simultaneously in an incubator? ICH guideline was not followed while carrying out stability studies.

I agree with you. Actually, it was a typing mistake. Now the sentence is revised accordingly.
Materials and Methods: Line 95, “Three samples were taken for initial analysis”, however, in Abstract 4 types of samples were mentioned (eg. T1, T2, T3 and T4). Which version is correct (3 or 4)?

Actually, we developed 4 different formulas (T1, T2, T3, AND T4). T4 was selected for further analysis. Analysis was repeated on 3 samples which were taken from T4. Now the stamen is revised to clarify this issue.

Materials and Methods: Line 100, Table 1, Procedure for preparing 1 % solution of Sitagliptin Phosphate solution was not correct.

The procedure is now revised and corrected accordingly
Materials and Methods: Line 102, “Five gram” precisely of the drug was dissolved in 30 mL distilled water. Whereas, in Table 1, 6.4 g was mentioned.

Thank you very much for this notification. I agree with you and the procedure is now revised accordingly.
Materials and Methods: There was no mention of mobile phase ratio in the entire manuscript, which is primary requirement for method to reproduce.

Now the mobile phase ratio is included

Materials and Methods: The method sample preparation was not satisfactory

Now the sample preparation is revised and changed appropriately.

Materials and Methods: Authors have not mentioned procedure for performing validation parameters, therefore, it is not possible to judge whether ICH guidelines were followed or not.

The procedure is now included
Results: Line 185, The pH was mentioned as “3 ± 0.15”, whereas, in Table 2, pH was in the range of 3-4.

Thank you again for this notification. Now the numbers are adjusted accordingly.
Results: Presentation of data and statistical analysis are not as per regulatory guidelines. Data’s presented in Table 2, 4 and 5 are not as per ICH guidelines

Done
Results: Chromatograms presented at the end of the manuscript are not visible properly and does not reflect the result or outcome of the study.

Better resolution chromatogram are now included

**Comment sheet**

Que.1: How you have selected a different formulation ingredients and ratio?

Thank you for your comment. Actually, we followed the quality by testing method which is now reported in the methodology.

Que.2: Have you taken UV Spectrum for selection of wavelength in SiP solution? Is there same or different?

Yes, they are same. Now we included this in the methodology and instrument sections.

Que.3: Which guideline follow for the evaluation of stability studies of SiP solution?

We followed the ICH guidelines for stability testing of pharmaceutical products and now this statement is included in the methodology.

Que.4: How you have evaluated organoleptic properties of SiP oral liquid formulation?

The organoleptic properties were evaluated by the formulator himself during the development phase. This was approved by the local ethics committee (Institutional Review Board [IRB] of An-Najah National University). This statement is now included in the methodology

Que.5: Have you develop HPLC method or cite any other research article method? Describe it.

The HPLC method was developed and validated by the team according to the ICH guidelines.

Reviewer #2: Research article should be recommended after such correction. Author doing good research work in the area of pharmaceutical formulation development and for the same he/she also develop a good HPLC method.

I would like to thank the reviewer for this positive comment and for the efforts that he/she did to improve the quality of the manuscript. We tried our best to answer the comments and to modify the manuscript accordingly. We believe that the manuscript is now much better and much closer to the final acceptance.

Best Regards
