# Supplementary material for: Stability of extemporaneously prepared sitagliptin phosphate solution
Source: PLoS One. 2022 Mar 16;17(3):e0262068. doi: 10.1371/journal.pone.0262068 (PMC8926268; doi:10.1371/journal.pone.0262068)
Supplement: Attachment — Submitted filename: PLOS review.docx [file pone.0262068.s003.docx]

Dear Editor

Thank you for selecting me as a reviewer. I tried my level best to increase the quality of this paper by making following suggestions.

Thanking you,

Yours faithfully

Sherin A

Reviewer

Comments: -

1. Sitagliptin is a dipeptidyl peptidase enzyme inhibitor which produce hypoglycemic along with diet and exercise to control sugar level in type 2 Diabetic Patients.

- But according to America diabetic Association best therapy for type 2DM in patient with feeding tubes or patients with swallowing difficulty is insulin therapy is good.

2. Is there any references regarding the Assay of sitagliptin tablets or sitagliptin phosphate powder?
3. Please provide information regarding Diluents used to prepare the stock solution.
4. Preparation of stock, sample and standard solutions for HPLC are not clear.
5. The sample T4 has a final concentration of 1%w/v. can you achieve 1% w/v if you dissolve 6.4 gm of SiP in 500 ml of water.
6. Is there any hypoglycemic effect has possessed by the sorbitol and mannitol? If it is there how you can suggest such a formulation. Please justify your suggestion
7. Each 100 ml of SiP formulation contain 2.8 gm of sorbitol, which higher than the concentration of active pharmaceutical ingredient SiP (1.28 gm in 100ml). Can you justify this with above comment no.5.
8. Regarding the sample solution preparation, author has to mention how you prepared the sample solution from the formulation (T4) you made.
9. How you read the colony forming units after the microbial contamination test.
10. How you did the assay of SiP T4 formulation? If you did the assay after the validation or be
11. There is a study (IJPSR, 2013; Vol. 4(9): 3494-3503- <http://citeseerx.ist.psu.edu/viewdoc/download?doi=10.1.1.414.784&rep=rep1&type=pdf> ) related with degradation products of Sitagliptin. Are you correlated your study with the previous study? Please include your comments in the discussion.
12. Please take some more time to rewrite the methods, report, discussion and conclusion. It would be a better paper. I strongly suggest you to rewrite these portions.

The end.
