# Supplementary material for: Stability of extemporaneously prepared sitagliptin phosphate solution
Source: PLoS One. 2022 Mar 16;17(3):e0262068. doi: 10.1371/journal.pone.0262068 (PMC8926268; doi:10.1371/journal.pone.0262068)
Supplement: Attachment — Submitted filename: To PLOS.docx [file pone.0262068.s005.docx]

Dear Editor,

Thank you for giving me a chance to review this article.

I am sorry that I could not finish the review on the actual date because I was suffering from Covid 19 and related health issues.

1. The author made changes according to previous comments.
2. Some references not following the PLOS guidelines for referencing. The author should recheck the Reference style.
3. In table no.1, “purified water up to 500 ml” shall be rewritten as purified water up to 640 ml to produce 1% w/v concentration.

The end
