# Supplementary material for: Stability of extemporaneously prepared sitagliptin phosphate solution
Source: PLoS One. 2022 Mar 16;17(3):e0262068. doi: 10.1371/journal.pone.0262068 (PMC8926268; doi:10.1371/journal.pone.0262068)
Supplement: Attachment — Submitted filename: Response Letter.docx [file pone.0262068.s006.docx]

Dear Editor,

Thank you for giving us a chance to revise our manuscript. Many thanks for you and respected reviewers for their efforts in improving our manuscript.

Reviewer comments

1. The author made changes according to previous comments.

Thank you a lot

2. Some references not following the PLOS guidelines for referencing. The author should recheck the Reference style.

Answer: Thank you and now we corrected references as shown in the revised MS and we followed Plos One style.

3. In table no.1, “purified water up to 500 ml” shall be rewritten as purified water up to 640 ml to produce 1% w/v concentration.

Answer: Thank you for this suggestion and for all your efforts in reviewing our manuscript and now we corrected Table 1 as you recommended

Best Wishes
