# Supplementary material for: Stability of extemporaneously prepared sitagliptin phosphate solution
Source: PLoS One. 2022 Mar 16;17(3):e0262068. doi: 10.1371/journal.pone.0262068 (PMC8926268; doi:10.1371/journal.pone.0262068)
Supplement: Attachment — Submitted filename: Response to the reviewer comments.docx [file pone.0262068.s004.docx]

**Dear Editor,**

Many thanks for providing us with the opportunity to revise our manuscript. We would also like to thank yourself and the reviewer for the time and expertise in providing feedback.

We think that all comments raised by the reviewer are legitimate and requires consideration. We would like to profoundly thank hiim for his constructive comments which have greatly improved the manuscript.

Please find below our response to reviewer comments. We have considered carefully all of the comments and have amended the manuscript as appropriate. The amended text is highlighted in red font throughout the manuscript.We have provided a detailed response to each of the comments.

Thanking you,

Yours faithfully

Comments: -

1. Sitagliptin is a dipeptidyl peptidase enzyme inhibitor which produce hypoglycemic along with diet and exercise to control sugar level in type 2 Diabetic Patients.

- But according to America diabetic Association best therapy for type 2DM in patient with feeding tubes or patients with swallowing difficulty is insulin therapy is good.
- Corrected as requested

2. Is there any references regarding the Assay of sitagliptin tablets or sitagliptin phosphate powder?

No reference was found as well as we used a new method.

3. Please provide information regarding Diluents used to prepare the stock solution.

We provided information regarding Diluents as requested.

4. Preparation of stock, sample and standard solutions for HPLC are not clear.

Thank you for this suggestion and now we clearly explain the preparation of stock, sample and standard solutions for HPLC.

5. The sample T4 has a final concentration of 1%w/v. can you achieve 1% w/v if you dissolve 6.4 gm of SiP in 500 ml of water.

Yes since we used sitagliptin in its salt form and not as free base.

6. Is there any hypoglycemic effect has possessed by the sorbitol and mannitol? If it is there how you can suggest such a formulation. Please justify your suggestion.

Actually, these two are polare excipients and have very limited impact on sugar blood level.

https://jums.ac.ir/dorsapax/Data/sub_7/file/Handbook%20of%20pharmaceutical%20excipients.pdf

<https://www.mayoclinic.org/diseases-conditions/diabetes/expert-answers/artificial-sweeteners/faq-20058038>

Ohrem HL, Schornick E, Kalivoda A, Ognibene R. Why is mannitol becoming more and more popular as a pharmaceutical excipient in solid dosage forms?. Pharmaceutical development and technology. 2014 May 1;19(3):257-62.

7. Each 100 ml of SiP formulation contain 2.8 gm of sorbitol, which higher than the concentration of active pharmaceutical ingredient SiP (1.28 gm in 100ml). Can you justify this with above comment no.5.

Sorbitol is an artificial sweetner and its concentration is within the recommended level reported in the handbook of pharmaceutical excipients

<https://jums.ac.ir/dorsapax/Data/sub_7/file/Handbook%20of%20pharmaceutical%20excipients.pdf>

8. Regarding the sample solution preparation, author has to mention how you prepared the sample solution from the formulation (T4) you made.

Done as requested.

9. How you read the colony forming units after the microbial contamination test.

By visual inspection using the colony counter as reported in the revised manuscript

10. How you did the assay of SiP T4 formulation? If you did the assay after the validation or be

It is now reported in the revised manuscript

11. There is a study (IJPSR, 2013; Vol. 4(9): 3494-3503- <http://citeseerx.ist.psu.edu/viewdoc/download?doi=10.1.1.414.784&rep=rep1&type=pdf> ) related with degradation products of Sitagliptin. Are you correlated your study with the previous study? Please include your comments in the discussion.

The comments are now reported in the discussion

12. Please take some more time to rewrite the methods, report, discussion and conclusion. It would be a better paper. I strongly suggest you to rewrite these portions.

All the required sections are rechecked and revised thoroughly as suggested.
